# Exploring mechanisms of action in clinical trials of complex surgical interventions using mediation analysis

**DOI:** 10.1177/1740774520947644

**Published:** 2020-08-20

**Authors:** Linda Sharples, Olympia Papachristofi, Saleema Rex, Sabine Landau

**Affiliations:** 1 Department of Medical Statistics, London School of Hygiene and Tropical Medicine, London, UK; 2 Novartis Pharma AG, Basel, Switzerland; 3 University of Sheffield, Sheffield, UK; 4 King’s College London, London, UK

**Keywords:** Complex surgical interventions, surgeon effects, clustering, treatment effect heterogeneity, co-interventions, mediation analysis, mediation estimands

## Abstract

**Background:**

Surgical interventions allow for tailoring of treatment to individual patients and implementation may vary with surgeon and healthcare provider. In addition, in clinical trials assessing two competing surgical interventions, the treatments may be accompanied by co-interventions.

**Aims:**

This study explores the use of causal mediation analysis to (1) delineate the treatment effect that results directly from the surgical intervention under study and the indirect effect acting through a co-intervention and (2) to evaluate the benefit of the surgical intervention if either everybody in the trial population received the co-intervention or nobody received it.

**Methods:**

Within a counterfactual framework, relevant direct and indirect effects of a surgical intervention are estimated and adjusted for confounding via parametric regression models, for the situation where both mediator and outcome are binary, with baseline stratification factors included as fixed effects and surgeons as random intercepts. The causal difference in probability of a successful outcome (estimand of interest) is calculated using Monte Carlo simulation with bootstrapping for confidence intervals. Packages for estimation within standard statistical software are reviewed briefly. A step by step application of methods is illustrated using the Amaze randomised trial of ablation as an adjunct to cardiac surgery in patients with irregular heart rhythm, with a co-intervention (removal of the left atrial appendage) administered to a subset of participants at the surgeon’s discretion. The primary outcome was return to normal heart rhythm at one year post surgery.

**Results:**

In Amaze, 17% (95% confidence interval: 6%, 28%) more patients in the active arm had a successful outcome, but there was a large difference between active and control arms in the proportion of patients who received the co-intervention (55% and 30%, respectively). Causal mediation analysis suggested that around 1% of the treatment effect was attributable to the co-intervention (16% natural direct effect). The controlled direct effect ranged from 18% (6%, 30%) if the co-intervention were mandated, to 14% (2%, 25%) if it were prohibited. Including age as a moderator of the mediation effects showed that the natural direct effect of ablation appeared to decrease with age.

**Conclusions:**

Causal mediation analysis is a useful quantitative tool to explore mediating effects of co-interventions in surgical trials. In Amaze, investigators could be reassured that the effect of the active treatment, not explainable by differential use of the co-intervention, was significant across analyses.

## Introduction

Large traditional randomised controlled trials (RCTs) of drug therapies, with rigorously controlled design, influence clinical practice (see, for example, Pocock).^1^ However, they may lack generalisability to the intended setting and for trials of surgical techniques, a degree of flexibility is required.^2–4^

RCTs in surgery are increasingly used in a range of disease populations, including cancer, orthopaedic and cardiac patients.^5–7^ These trials often adopt pragmatic designs to reflect the intervention as performed in clinical practice. During trial design, decisions are made about the inclusion and level of standardisation of each stage of the procedure, rather than imposing a strictly standardised protocol.^8^ The technically demanding nature of surgery, requirements of individual patients and unexpected adverse events result in flexibility of delivery. A related issue is the use of co-interventions alongside or subsequent to the surgery under investigation which, although not part of the intervention, may impact outcomes and mediate the treatment effect.

In RCTs, primary analysis typically assesses effectiveness of the whole surgical procedure in the ‘Intention to Treat’ population. However, there may be interest in exploring contributions to the overall treatment effect of intervention components, patient characteristics, surgeons or co-interventions. Some variables are effect modifiers, modelled as fixed effects and interactions, others are not of interest themselves, but introduce some dependency (clustering) between trial participants. For example, outcomes for cardiac surgeons are clustered, even after adjusting for patient characteristics and may be analysed as random effects.^9–11^ An analogous situation exists in psychology where clustering of patient outcomes by therapist is expected.^12^ Co-interventions are not part of the intervention package but may affect trial results, especially if they are not applied consistently across trial arms. Co-interventions may mediate the effect of the intervention so that secondary analysis could explore the extent to which the surgery acts directly on the outcome and indirectly via the co-intervention. Furthermore, policy makers may be interested in the direct effect of the surgery if (hypothetically) either all or no patients receive the co-intervention.

Causal mediation analysis has been used extensively to explore how complex interventions work in other contexts, notably in psychotherapy, where the mediated (indirect) effect of treatment is usually the focus.^13^ In contrast, for surgical trials, interest centres on direct effects of the intervention; the co-intervention is a nuisance mediator and we wish to exclude its effect. Rigorous statistical methods for assessing causal relationships have been developed, but uncertainty remains on when they are relevant in this setting; applications to real trials would help to clarify their usefulness. In the surgical trials literature, few studies have considered mediation in trial analysis and none where both mediator and outcome are binary, or where clustering of outcomes is apparent.^14,15^

The focus of this article is primarily on defining relevant causal estimands in surgical trials, specifying statistical approaches for estimating these and being clear about the assumptions made when doing so. Methods are illustrated using the Amaze heart surgery trial.^7,16^

We provide background, methods and assumptions for causal mediation methods based on counterfactual arguments, and an overview of resources for implementation of methods in standard statistical software in the section ‘Methods’. These are applied to the Amaze trial, which motivated this work as described in the section ‘Results’; conclusions and discussion are provided in the section ‘Conclusion’.

## Methods

**Figure 1.**
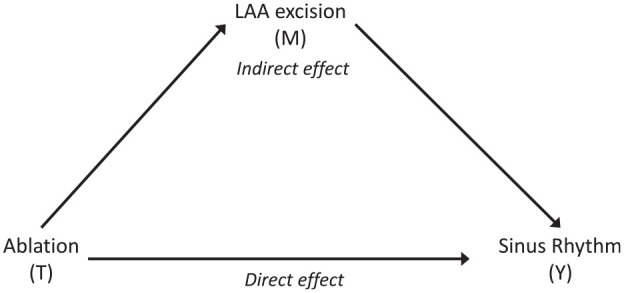
Simple causal graph for the Amaze trial assuming no confounding. LAA: left atrial appendage.

Mediation analysis aims to explain treatment mechanisms by partitioning the total effect of an intervention on an outcome into direct effects and indirect effects, which act via a mediator (see, for example, study by MacKinnon^13^ and Figure 1). What distinguishes a mediator from a moderator is that it occurs after randomisation and lies on the causal pathway between intervention and outcome.^17^ A major difficulty in this context is the presence of unexplained confounding of the mediator–outcome path which, if ignored, results in biased estimates of direct and indirect effects of the intervention.

Since the landmark publications of Baron and Kenny^18^ and Robins and Greenland^19^, use of mediation analysis has increased substantially. There is extensive literature on mediation in RCTs of psychotherapy, but from a targeted literature search there has been little uptake in the analysis of mechanisms in surgical trials.^12^

Early publications focussed on the case where either mediator or outcome were continuous random variables and based analyses on linear models.^18,19^ Subsequent developments provided more general methods, including conditions for identifiability of estimands.^20,21^ Methods for more complex situations are being developed with recent literature considering multiple mediators and repeated exposures (interventions) with repeated mediators.^22–24^ Moreover, methods have been developed within the Bayesian paradigm.^25^ In this study, we focus on a single (binary) mediator and a single (binary) outcome, where the main estimand of interest is the direct difference between trial arms in the probabilities of a successful outcome, that is not due to confounding by a nuisance mediator. Several authors provided methods for estimation of effects when both mediator and outcome are binary so that new methods for our case are not required.^26^ In this context, we briefly review the framework and conditions required to estimate causal estimands of interest in the presence of a single mediator.

### Potential outcomes, natural direct, natural indirect and controlled direct effects

In the counterfactual approach, a number of potential outcomes may be considered, depending on the treatment (surgery) and mediator (co-intervention) levels.^27^ We denote the potential outcome for participant i,i=1,…,n, who received treatment t∈(0,1) and mediator m∈(0,1) by $Y_{i}(t,m)$. The potential mediator for patient *i* under treatment *t* is expressed as $M_{i}(t)$. In our case, for each trial arm, there are two potential values for the mediator, $M_{i}(1)$ and $M_{i}(0)$, resulting in four potential outcomes (two treatment arms × two mediator levels); only one of these is observed for each participant, so that there are one factual and three counterfactual outcomes. For a patient to be eligible for mediation analysis, all four must be possible (theoretically). In practice, this assumption is difficult to assess from trial data alone unless suitability for the mediator irrespective of treatment arm is recorded prospectively or available from operative notes, and must be evaluated in discussion with participating surgeons.

The total causal effect of the treatment on patient *i*’s outcome is defined as $Y_{i}(1,M_{i}(1))-Y_{i}(0,M_{i}(0))$. The trial analysis focusses on the total average causal intervention effect

$$E[Y_{i}(1,M_{i}(1))-Y_{i}(0,M_{i}(0))].$$

Since treatment allocation is independent of potential outcomes in RCTs $\left(Y_{i}(1),Y_{i}(0)╨T_{i}\right)$, the total average causal effect of the intervention can be identified using the data from each trial arm.

Following the study by Imai et al.,^21^ we provide expressions for natural direct, natural indirect and total effects for a patient *i* in Table 1. The natural indirect effect $\delta_{i}(t)$ represents change in the outcome if the mediator for patient *i* was changed from its value for the intervention arm ($M_{i}(1)$) to its value in the control arm ($M_{i}(0)$), while holding treatment arm constant at **t**, for t=0,1. The natural direct effect $\zeta_{i}(t)$ represents the effect of intervention *t* on outcome, holding the mediator at the level realised under *t*. Because our chosen estimand is the difference in probability of successful outcomes between trial arms, these two expressions can be combined to obtain the total natural effect for patient *i*. Note that this would not be the case had we chosen the relative risk or odds ratio as the estimand.

**Table 1.** Definitions of natural direct, natural indirect, total and controlled direct effects for an individual patient *i*.

Algebraic specification	Definition
$\begin{array}{c} \delta_{i}(1)\equiv Y_{i}(1,M_{i}(1))-Y_{i}(1,M_{i}(0))\\ \delta_{i}(0)\equiv Y_{i}(0,M_{i}(1))-Y_{i}(0,M_{i}(0))\\ \delta_{i}(a*)\equiv (\delta_{i}(1)+\delta_{i}(0))/2 \end{array}$	Natural indirect effect for the intervention, control arms and their average actingthrough the mediator
$\begin{array}{c} \zeta_{i}(0)\equiv Y_{i}(1,M_{i}(0))-Y_{i}(0,M_{i}(0))\\ \zeta_{i}(1)\equiv Y_{i}(1,M_{i}(1))-Y_{i}(0,M_{i}(1))\\ \zeta_{i}(a*)\equiv (\zeta_{i}(1)+\zeta_{i}(0))/2 \end{array}$	Natural direct effect of treatment fixing the mediator at the control, intervention or average level
$\begin{array}{c} \tau_{i}\equiv Y_{i}(1,M_{i}(1))-Y_{i}(0,M_{i}(0))\\ =\delta_{i}(1)+\zeta_{i}(0) \end{array}$	The total natural effect (TE)
$\begin{array}{c} \zeta_{i}^{c}(1)\equiv Y_{i}(1,1)-Y_{i}(0,1)\\ \zeta_{i}^{c}(0)\equiv Y_{i}(1,0)-Y_{i}(0,0) \end{array}$	Controlled direct effect of treatment if mediator is mandated or prohibited

a* denotes the average of the two treatment arms as the reference.

For probability-difference estimands, natural direct and indirect effects from the trial are defined as the expectation of patient-specific effects over trial participants. Note that causal effects are defined at a given reference level for the mediator, often set to the level observed in the control arm or intervention arm or the average of the two (see Table 1). All these causal mediation estimands can be estimated from trial results provided that identifiability assumptions hold (see below).

In some applications, interest centres on estimating either the contribution of the mediator to the total effect, or, as in our example, the causal direct effect after taking the mediator into account. Some mediators can be considered manipulable in that their application could be controlled (at least in theory).^28^ For example, surgeons and policy makers may be interested in the effect of the intervention if the co-intervention (mediator) was either mandated or prohibited. This (hypothetical) question can be addressed by re-estimating effects with the mediator level set to 1 (or 0) for all cases (see Table 1 for notation).^29^

Fundamental to the potential outcomes approach is the stable unit treatment value assumption, which has two parts.^30^ First, it assumes no interference between patients, in that potential outcomes for a patient are not affected by treatments that other patients receive. Second, it assumes consistency, in that there are no versions of the treatment that can lead to different potential outcomes.

Additional assumptions are required in order to identify, and interpret causally, the mediation effects.

Imai and colleagues show that average causal mediation effects are identifiable in general (without other distributional assumptions) providing the key assumption of Sequential Ignorability holds.^31^ This assumption requires two conditions:

1. $Y_{i}(t,m),M_{i}(t)╨T_{i}|X_{i}$, given baseline variables and other confounders (including random effects), treatment assignment is independent of potential outcomes and mediators.
2. $Y_{i}(t,m)╨M_{i}(t)|T_{i}=t,X_{i}$, given observed treatment, baseline variables and other confounders (including random effects), the mediator assignment is independent of potential outcomes.

The first condition is justifiable in RCTs since random allocation is independent of subsequent events, including mediators and outcome measurements. Randomisation protects intervention–mediator and intervention–outcome relationships from confounders. The second condition is difficult to justify and not testable from observed data. Randomisation does not protect the mediator–outcome relationship from confounding because those with high observed mediator levels can differ from those with low values on prognostic variables. Because bias due to unmeasured confounding cannot be excluded in general, it is important to conduct sensitivity analysis to assess this assumption.

Sequential Ignorability is also required for causal interpretation of controlled direct effects, as is the assumption that all patients have the potential to receive all treatments and mediator levels. Controlled effects estimation also requires that the mediator is manipulatable.

### Resources for estimation of direct and indirect effects

Natural direct and indirect effects can be estimated in standard statistical software. We describe commonly cited examples, although contributions continue to be published. The user-friendly *mediation* package in *R* uses Monte Carlo simulation to estimate direct and indirect effects on the additive scale for continuous and binary mediator and outcome variables, and includes both parametric and non-parametric error options.^32^ This package accommodates intervention–mediator interaction, random effects and sensitivity analysis for unobserved confounders, although sensitivity analysis when both mediator and outcome are binary is not incorporated. It provides estimates of difference in proportions estimands but not odds ratio estimands. A limited version of this package for parametric estimation is programmed in *Stata*.^33^ Other *R* packages are available for multiple mediators (*mma*) and for estimands on odds ratio scale (*medflex*).^34,35^

Alternative *Stata* packages are available for parametric estimation (*paramed*), for binary outcomes with multiple mediators (*LDEcomp*) and for estimating marginal distributions for time-varying exposure (intervention) and covariates (*gformula*).^36–38^ The *gformula* package allows continuous and binary mediators and outcomes, intervention–mediator interactions and options for missing data; *gformula* is also available as a *SAS* macro. A fully parametric procedure for mediation analysis is available in both *SAS* and *SPSS* (*CAUSALMED*).^39^

For analysts with a thorough understanding of do-calculus and directed acyclic graphs, Tikka and Karvanen^40^ contributed the *R* package *causaleffect*.

## Results

The Amaze cardiac surgical trial assessed whether ablation during heart surgery returns the heart to normal sinus rhythm in patients with a documented history of rapid or irregular heart rhythm.^7,16^ This multi-centre, Phase III, pragmatic RCT randomised 352 patients to ablation plus planned surgery, or planned surgery alone (control arm). The primary outcome was sinus rhythm restoration at one year post-surgery (binary outcome).

In 280 trial patients with valid primary outcome, 84/137 (61.3%) ablation and 67/143 (46.9%) control patients returned to sinus rhythm. Of 151 patients with a successful outcome at one year, 84 (56%) were in the ablation arm, 48 (32%) were in sinus rhythm during a baseline electrocardiograph despite having a history of atrial fibrillation and mean (SD) age was 70.5 (8.0) years. Of 129 patients with an unsuccessful outcome, 53 (41%) were in the ablation arm, 8 (6%) were in sinus rhythm at baseline and mean (SD) age was 73.6 (7.0) years.

The original trial analysis using mixed effects logistic regression, including baseline fixed effects (heart rhythm at baseline, patient age and cardiac operation type) and surgeon random effects are in Table 2 (results for operation type suppressed for simplicity). The odds of successful outcome were higher in the ablation arm, for younger patients and for those in sinus rhythm at baseline. Adjusting for fixed effects, 8.4% of the remaining variation in outcomes was due to surgeon effects. We used Monte Carlo simulation to obtain our chosen estimand (difference in proportion of patients in normal heart rhythm at one year), estimated to be 0.17 (0.06, 0.28); that is, 17% more ablation patients returned to sinus rhythm than did control patients. Note that this is a marginal estimand, as opposed to the odds ratio for ablation which is conditional on other variables in the parametric model.

**Table 2.** Estimated odds ratios for return to sinus rhythm at one year using data from the Amaze trial (results for operation type are suppressed).

	Original trial analysis	Outcome model for mediation
Variable	Odds ratio (95% CI)	Odds ratio (95% CI)
Ablation	2.43 (1.40, 4.21)	1.59 (0.76, 3.32)
LAA removal		0.75 (0.30, 1.84)
Ablation–LAA interaction		2.57 (0.78, 8.44)
Baseline sinus rhythm	8.31 (3.42, 20.20)	8.58 (3.50, 21.06)
Age in years	0.96 (0.92, 1.00)	0.96 (0.92, 1.00)
ICC(surgeon)^a^	0.084	0.102

CI: confidence interval; LAA: left atrial appendage.

a ICC(surgeon) is the Intra-Cluster Correlation Coefficient due to surgeon random effects on the log-odds scale, calculated as the proportion of total variation attributed to variation between surgeons. Level 1 residual variance is $\sigma_{e}^{2}=\pi^{2}/3$ using the latent variable formulation of the logistic regression model.

The heart contains a sac called the left atrial appendage (LAA) in which blood clots can form. Although not a component of routine cardiac surgery or ablation, some patients had the LAA removed during surgery (97 (55.1%) of 176 patients who had ablation and 53 (30.1%) of 176 control patients). The difference in the probability of LAA removal between trial arms raised concerns that some of the observed total effect of ablation may have resulted from this co-intervention.

Using the potential outcomes framework, we explored the relative size of the direct effects of ablation on the probability of returning to sinus rhythm and an indirect effect acting through LAA removal, see Figure 1. In particular, it is important to ensure that a significant proportion of the treatment effect resulted directly from ablation.

In addition to ‘Intention To Treat’ analysis, we explore questions such as:

- How much of the intervention effect acts as a direct effect of ablation, rather than through removal of the LAA?
- What would the effect of ablation be if no patient in the target population (or all patients) had the LAA removed?
- Does the direct effect of ablation vary between patients and how?

### Natural direct, natural indirect and controlled direct effects in the Amaze trial

Although our estimand of interest is the difference between trial arms in probability of sinus rhythm restoration, in keeping with the original modelling approach we used mixed effects logistic regression models to describe the relationships between outcomes, mediators and treatment, adjusting for confounders. Two additional parametric models are required for this purpose, the mediator model and the outcome model. Our mediator model was

$$\mathrm{logit}(p(M_{i}|T_{i},X_{i},v_{s_{i}})=\alpha_{0}+\alpha_{1}T_{i}+\alpha_{2}^{T}X_{i}+v_{s_{i}}$$

where for patient i=1,…,n, $T_{i}$ and $M_{i}$ represent treatment assignment and observed LAA removal status, respectively; $X_{i}$ represents baseline covariates; and $v_{s_{i}}$ surgeon random effects with $v_{s_{i}}|T_{i},X_{i}\sim N(0,\sigma_{v}^{2})$.

The outcome model was

$$\begin{array}{c} \mathrm{logit}(p(Y_{i}=1|T_{i},M_{i},X_{i},u_{s_{i}}))=\theta_{0}+\theta_{1}T_{i}+\theta_{2}M_{i}\\ \;+\theta_{3}(T\times M{)}_{i}+\theta_{4}^{T}X_{i}+u_{s_{i}} \end{array}$$

where $Y_{i}$ is the binary outcome, $(T\times M{)}_{i}$ denotes the interaction between treatment and mediator and $u_{s_{i}}|T_{i},M_{i},X_{i}\sim N(0,\sigma_{u}^{2})$ for surgeon effects. This differs from the overall trial analysis by adjustment for LAA removal and its interaction with treatment.

Estimation of the natural direct effect of ablation on return to sinus rhythm and the natural indirect effect of ablation via removal of the LAA can be estimated from these equations using either approximate methods or by Monte Carlo simulation.

Table 2 summarises the outcome model results alongside the original trial analysis. The coefficient for ablation decreased substantially when LAA removal and its interaction were included in the model; the (control group) mediator was associated with a small, non-significant increase in the odds of a successful outcome. Older patients were less likely to have a successful outcome, while those in sinus rhythm at baseline had much greater chance of returning to sinus rhythm.

Table 3 shows that removal of the LAA was strongly associated with intervention (ablation) and age, with older patients less likely to have the LAA removed. The intra-cluster correlation coefficient was very high (56%), suggesting that individual surgeons had strong preferences for removal (or not) of the LAA. All analyses use complete cases only and, according to the sequential ignorability assumption, we assume that age, baseline sinus rhythm, operation type and surgeon comprise all important confounding variables for the LAA-–outcome association.

**Table 3.** Estimated odds ratios for LAA removal for the mediator model using data from the Amaze trial (results for operation type suppressed).

Variable	Odds ratio (95% CI)
Ablation	4.78 (2.65, 8.64)
Baseline sinus rhythm	0.51 (0.23, 1.16)
Age in years	0.94 (0.90, 0.99)
ICC (surgeon)^a^	0.56

LAA: left atrial appendage; CI: confidence interval.

a ICC(surgeon) is the intra-cluster correlation coefficient due to surgeon random effects on the log-odds scale, calculated as the proportion of total variation attributed to variation between surgeons. Level 1 residual variance is $\sigma_{e}^{2}=\pi^{2}/3$ using the latent variable formulation of the logistic regression model.

#### How much of the intervention effect acts as a direct effect of ablation, rather than through the removal of the LAA?

To address this question, we used the *R* package *mediation*; since mediator and outcome are binary, it allows random effects models and estimates the difference in probability of success, our chosen estimand.^32^ To estimate the probabilities of success, first 2000 potential mediators for each treatment arm were simulated from the logistic model for mediator; then, conditional on each treatment × simulated–moderator pair, potential outcomes were simulated from the logistic model for outcome. Probabilities of success for each treatment were estimated from these simulated samples. The non-parametric (Bootstrap) option was used for inference (see Appendix for details of the algorithm).

**Figure 2.**
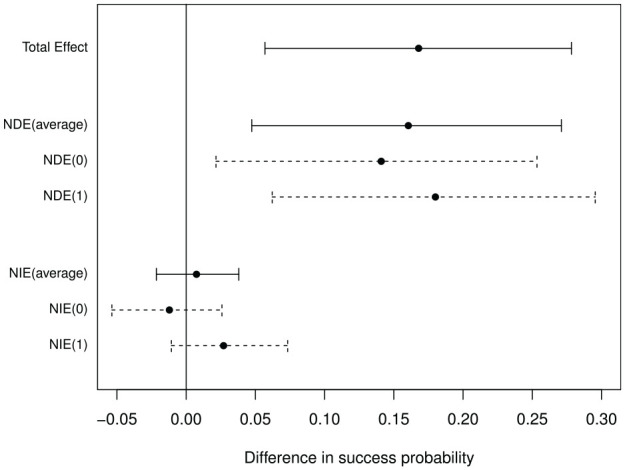
Total effect (TE), natural direct effects (NDE) and natural indirect effects (NIE) of ablation on return to normal heart rhythm (with mediator at the level of control arm (0), intervention arm (1) and the average).

Total effect of ablation on probability of return to sinus rhythm was 0.17 (0.06, 0.28); that is, 17% more ablation patients than controls returned to sinus rhythm, (95% confidence interval: 6%, 28%). Figure 2 shows that, when LAA removal is averaged over intervention and control arm levels, about 1% (−2%, 4%) of the effect of ablation acts via removal of LAA, with 16% (5%, 26%) a direct effect of ablation. Results are similar when the reference level for LAA removal is set at either intervention or control arm levels.

Alternative parametric models for surgeon effects, lack of interaction between ablation and LAA removal, software packages and choice of parametric and non-parametric confidence intervals showed very similar results. In all models the natural direct effect was ‘statistically significant’ at the 1% level, so that the trial conclusions were confirmed.

#### What would be the effect of ablation if no patient in the target population (or all patients) had the LAA removed?

Setting the mediator level to 0 (1) for all cases and estimating controlled effects, the difference in the percentage of patients with a successful outcome due to ablation was 14% (2%, 25%) if nobody had the LAA removed, and 18% (6%, 30%) if everyone had the LAA removed. LAA removal may have a small but important impact on the effectiveness of ablation.

These results hold if all variables affecting the decision to remove the LAA have been adjusted for; in reality, there may be systematic selection of patients for LAA removal and some unmeasured confounding.

#### Does the direct effect of ablation vary between patients and how?

**Figure 3.**
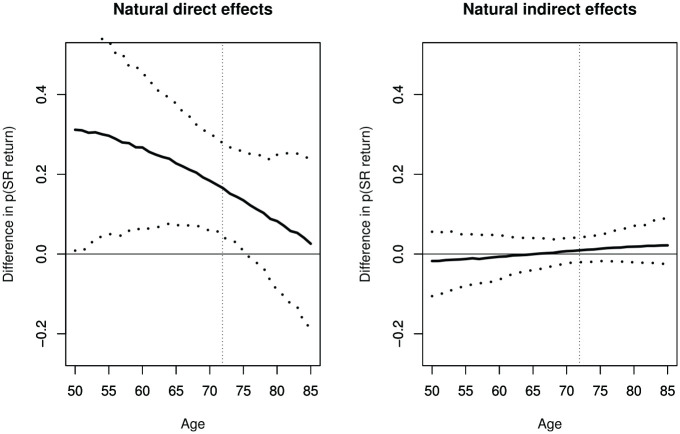
Estimated natural direct effect (left) and natural indirect effect (right), with 95% confidence intervals, as a function of patient age (SR: sinus rhythm; vertical line at mean age of trial sample 71.9 years).

Model results and clinical colleagues suggested that LAA removal and return to sinus rhythm are age-related. The moderating effect of age on total and mediated effects was explored, by including age and its interactions with ablation and LAA removal in the parametric models. The results in Figure 3 suggest that the direct (and total) effects decrease with age, but there is little evidence that mediation is associated with age.

### Sensitivity analysis

Assessing sensitivity of results to unobserved confounding is important. For continuous mediator and outcome, sensitivity is assessed through correlation between residual errors from mediator and outcome models, which is zero if sequential ignorability holds.^31^ If plausible correlation levels substantially change estimates of direct and indirect effects, uncontrolled confounding may be distorting true causal effects. The same approach can be used, if only one of mediator and outcome is continuous and the other binary (probit analysis).^31,32^

Vansteelandt in the Appendix to Benitez Majano^41^ provided methods to address unmeasured covariates when both mediator and outcome are binary, based on three parameters.

Define *U* to be a continuous measure encapsulating all unobserved confounders, scaled to have variance 1, and β the log (odds ratio) of *U* on outcome *Y*, conditional on *T* and *M*. To express how strongly *U* is associated with the mediator, define two further sensitivity parameters $\lambda_{0}$ and $\lambda_{1}$ such that

$$E(U|M=1,T=t)-E(U|M=0,T=t)=\lambda_{0}+\lambda_{1}t$$

That is, $\lambda_{0}$ represents the association between *U* and *M* in the control arm and $\lambda_{1}$ the additional association due to the intervention.

To explore sensitivity to unexplained confounding, β, $\lambda_{0}$ and $\lambda_{1}$ must be varied, with 0 representing no unexplained confounding and ±1 large effects. In our analysis, we address the question,

*How large do the parameters β, $\lambda_{0}$ and $\lambda_{1}$ have to be so that the mediation effect increases and the causal direct effect shrinks to zero?*

The *mediation* package in *R* was augmented to include sensitivity analysis when both mediator and outcome are binary (code available from authors).

A contour plot of natural direct effect for ablation by β and $\lambda_{0}$, ($\lambda_{1}$ set to 0 throughout), is provided in the Appendix. The direct effect of ablation was significant at the 5% level unless either of the following:

1. unexplained confounding had odds ratio for successful outcome of 2, and the confounder had expected value 0.5 standard deviations lower in patients with LAA removal, or
2. the unexplained confounding had odds ratio for successful outcome of <0.5, and the confounder has expected value 0.5 standard deviations lower in patients with LAA intact.

Therefore, unless unobserved confounding is strongly associated with both outcome and mediation, and acts in opposite directions, conclusions are unchanged. Neither scenario seems plausible.

## Conclusion

Mediation analysis is a useful tool to investigate questions of interest in RCTs provided key assumptions hold.^20^ Its use has been established in psychotherapy and other mental health trials but is uncommon in surgery.^29^

In Amaze, treatment success was observed in 17% more ablation patients than controls and, despite the strong association between ablation and LAA removal, the treatment effect was largely directly attributable to ablation. Although exploration of mediation effects could be limited by the size of the trial, this was not an issue in Amaze. In all models, the direct effect of ablation was significant (*p* < 0.01), even in a model that prohibited removal of the LAA. Sensitivity analysis suggested that unexplained confounding would have to be implausibly strong to conclude otherwise. Policy makers, surgeons and patients can be confident that ablation is effective in restoring sinus rhythm at one year, whatever the surgeon’s preference for the co-intervention.

In Amaze, 20% of patients did not have the primary outcome data, mostly due to death or measurement device failure. Since the proportion of missing cases was balanced in the two arms and missingness was unlikely to depend on intervention or mediator, complete case analyses were used. However, multiple imputation or inverse probably weighting methods for missing data could be incorporated into the analysis with additional programming.

Methodology for pragmatic trials of complex interventions has extended the use of RCTs for interventions like surgery. Flexibility in intervention delivery confers external validity and relevance of trial results, but introduces treatment heterogeneity. If there is too little control of treatment delivery, results are difficult to interpret and unlikely to be accepted by surgeons or policy makers. Therefore, detailed description of the substantive components of intervention and control, and monitoring of adherence to these protocols are crucial.^8,42^ Co-interventions should be considered at the design stage, with conditions for their use documented; additional baseline variables affecting the mediator–outcome relationship should be collected and included in analyses, to justify the assumption of sequential ignorability.

Causal mediation methods available in standard software cover a wide range of analyses, although assumptions must be considered carefully. Methods for multiple mediators, longitudinal outcomes, multiple treatments over time and time-to-event outcomes need further development.

Mediation analysis provides useful insights in trials of surgery that may lead to co-interventions and allows assessment of the potential size of their impact. Such quantitative assessments are a useful addition to qualitative process evaluations in RCTs.^43^

## Figures and Tables

**Figure 4.**
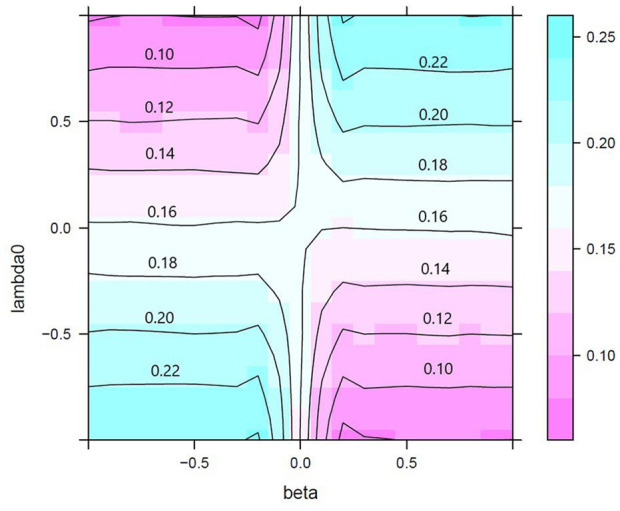
Contour plot of natural direct effect (average level of mediator) for the difference in probability of a successful outcome in the Amaze trial for varying values for β, the log odds ratio of the confounder on probability of success, and $\lambda_{0}$, the difference in mean confounder between mediated and non-mediated patients, with $\lambda_{1}$ set to zero.

## Appendix

### Estimation in the Amaze trial

For Amaze, we used the *R* package *mediation*, since mediator and outcome are binary, it allows random effects models and estimates the difference in probability of success.^32^ The non-parametric (Bootstrap) option was used for inference.

Following the study by Imai et al.,^21^ the algorithm took the following steps,

1. Sample *n* patients with replacement from trial dataset.
2. Using this sample fit the mediator model adjusted for baseline covariates.
3. Fit the outcome model including intervention, mediator, their interaction and confounders.
4. With the intervention set at t=0 and t=1 separately, simulate potential mediator values $M_{i}(0)$ and $M_{i}(1)$, for i=1,…,n from mediator model.
5. For each potential mediator, sample a single potential outcome $Y_{i}(t,M_{i}(0))$ and $Y_{i}(t,M_{i}(1))$ from the outcome model, for t=0,1.
6. From these n×4 potential outcomes sampled, calculate the total, direct and indirect effects (differences in probabilities) and average over all patients (see Table 1).
7. Repeat the above *J* times (2000 in the Amaze example) in order to compute summary statistics and non-parametric confidence intervals.

In the fully parametric version of this algorithm, mediator and outcome models are fitted to trial data, with estimated parameters $\hat{\alpha }$, $\hat{\theta }$, each assumed to have multivariate normal distributions. Values of α and θ are sampled from the fitted models, potential mediator and outcome samples are then generated conditional on α and θ and the algorithm follows the same steps as the non-parametric version thereafter.

### Sensitivity analysis results

In sensitivity analysis, total, natural direct and natural indirect effects were re-estimated for a range of values for β, the log (odds ratio) of the effect of unexplained confounders *U* (scaled to have variance 1) on the outcome *Y* and $\lambda_{0}$, the mean difference in *U* between mediated and non-mediated patients in the control arm. We set $\lambda_{1}$ to zero in all analyses, since it is unlikely that confounding associated with the mediator differs between treatment arms in our example.

Figure 4 is a contour plot of the estimated natural direct effect (average mediator level) for values of β and $\lambda_{0}$ ranging from −1 to 1. The difference in probability of success between the two arms remains above 0.1 (10%) unless β and $\lambda_{0}$ have opposite signs and are large. For example, this estimand only falls below 0.1 if,

1. the effect of the unexplained confounder on outcome is close to 1 (odds ratio 2) and has expected value at least 0.5 standard deviations lower in the LAA patients, or
2. the effect of the unexplained confounder on outcome is close to −1 (odds ratio <0.5) and has expected value at least 0.5 standard deviations lower in the non-LAA patients.

Moreover, the estimated difference in probability of success is significant at (at least) the 5% level for all other scenarios. For example, for the natural indirect effect of ablation to be overestimated by an amount that would change the conclusion, we would have to omit highly effective confounders that also have much higher levels in the non-LAA patients. The existence of such a confounder that is either unknown to investigators, or not considered important enough to measure and adjust for in analysis, seems implausible in an RCT. Therefore, conclusions concerning the effectiveness of ablation are unchanged for all plausible scenarios.
